# Conservation in the Andean Highlands of South America: A Habitat Enhancement Plan for *Tematobius philippii*, a Critically Endangered Species in the Ascotán Salt Flat in Chile

**DOI:** 10.3390/ani15213156

**Published:** 2025-10-30

**Authors:** Alejandra Alzamora, Hugo Salinas, Juan Carlos Trujillo, Gabriel Lobos

**Affiliations:** 1 Ecodiversidad Consultores, Santiago 8150000, Chile; alealzamora@yahoo.com (A.A.); hugo.salinas.m@gmail.com (H.S.); jctrujillo01@gmail.com (J.C.T.); 2 Centro de Gestión Ambiental y Biodiversidad, Facultad de Ciencias Veterinarias y Pecuarias, Universidad de Chile, Santiago 8820000, Chile

**Keywords:** amphibians, habitat enrichment, refuges, conservation

## Abstract

**Simple Summary:**

In the Ascotán salt flat, located in the north of Chile, lives a unique endemic frog called *Telmatobius philippii*. Its survival is threatened by human activity, partly due to industrial water use, and a lack of shelters. To aid the conservation of this endangered species, we created and installed artificial refuges—shelters made from clay tiles—in one of the springs. Over two years of monitoring, we found that frogs of all ages, from tadpoles to adults, quickly began using these man-made homes. The number of frogs using the shelters increased over time, and even eggs were found, showing successful breeding. This successful habitat improvement project provides a practical and promising strategy for conserving fragile species in areas impacted by human activity, offering a valuable tool for future conservation efforts.

**Abstract:**

Amphibians face a global conservation crisis, driven largely by habitat degradation. Effective and practical strategies for habitat restoration are urgently needed, particularly for Critically Endangered species in human-impacted ecosystems. *Telmatobius philippii* is a species classified as Critically Endangered by the IUCN. Its habitat is restricted to a few thermal springs in the Ascotán salt flat in Chile. A significant portion of one of these springs, V11, dried up in 2005 due to industrial groundwater withdrawals, leading to the loss of natural refuges and population decline. As part of a recovery plan for this spring we implemented a habitat improvement program by installing artificial refuges (clay tiles, bricks, and rock piles) and monitored their use over a two-year period. The results indicated that the refuges, particularly the clay tiles, were utilized by *T. philippii* at all life stages (larvae, juveniles, and adults). Refuge occupancy increased over time, reaching 75% by the end of the study, and the presence of eggs and early-stage larvae confirmed successful breeding associated with the artificial structures. This demonstrates the positive effect of artificial refuges as a practical tool for the recovery of *Telmatobius* populations. To our knowledge, this study provides the first documented case of successful habitat enhancement for this threatened group of high Andean amphibians, offering a replicable strategy for conservation in fragile ecosystems.

## 1. Introduction

Amphibians are a group of significant conservation concern due to their well-documented global decline [1,2,3]. The main threats include complex processes such as habitat destruction and fragmentation, changes in land use, climate change, ozone depletion, emerging diseases, chemical pollutants, overexploitation, and invasive species [4,5,6,7,8,9,10,11,12].

The genus *Telmatobius* Wiegmann, 1834, is found between 5° and 27° south latitude, from Ecuador to northern Argentina. Currently, 60 species are recognized [13], and it is considered one of the most threatened amphibian genera globally [14]. Most *Telmatobius* species are considered aquatic and are adapted to life under conditions of high elevations, intense solar radiation, and drought [15,16,17,18,19].

Seven species of *Telmatobius* have been reported in Chile [13], mainly associated with high-altitude environments [20,21,22]. *Telmatobius philippii*, Cuevas and Formas, 2002, was originally described in the ravines of Amincha and Del Inca at 4000 m a.s.l. in the Antofagasta Region, Chile. Subsequently, the populations inhabiting the Ascotán and Cárcote salt flats were reclassified as *T. philippii* based on genetic analysis [20,23].

The floodplains associated with the Ascotán springs are home to several endemic aquatic species such as the fish *Orestias ascotanensis* [24] and the snail *Heleobia ascotanensis* [25]. The main threats to aquatic life at this salt flat are groundwater extraction for metal mining and salt extraction [26]. Recently, the Ascotán salt flat was listed by the Chilean government as one of the exploitable salt flats for lithium, representing one threat to amphibian populations distributed within the so-called lithium triangle [27]. The commercial interest in lithium and boron in the Ascotán salt flat could adversely affect this complex and fragile ecosystem [28]. Recently, an integrated analysis of anthropogenic pressures and habitat availability prioritized this salt flat in terms of its protection, highlighting the presence of *T. philippii* [29].

In 2005, spring number 11 (V11) of the Ascotán salt flat was depleted due to groundwater extraction [26], which significantly reduced water levels in the lagoon and floodplain. Subsequent to this event, an artificial water recharge system was installed to divert groundwater from nearby wells to provide V11 with a continuous water supply [30]. However, one effect of the water depletion in V11 was the exposure of cavities and crevices along the bank walls that were previously submerged and provided refuges for *Telmatobius*. The refuges were also unintentionally damaged during subsequent activities associated with a floodplain revegetation program. A previous study by the National Regional Development Fund (Fondo Nacional de Desarrollo Regional—FNDR) titled “Diagnosis and Conservation of High Andean Amphibians, Antofagasta Region” [31], determined that the *Telmatobius* population in V11 was very small and demonstrated minimal evidence of successful reproduction (presence of larvae), and researchers concluded that the absence of natural refuges was a significant limiting factor for this isolated population. Almost all the frogs observed during that study were associated with one of the remaining refuges, a 60 cm cavity located under a small concrete flow metering structure located in the central part of the lagoon.

Considering that *T. philippii* requires refuges for reproduction (such as cavities and crevices), a recovery plan was proposed, which, to our knowledge, represents one of the first plans of this type. If refuge availability is a limiting factor for *Telmatobius*, we expect that these measures will contribute to improved reproductive success, thereby supporting the development of conservation plans for these threatened amphibians in South America. Regarding this, an artificial refuge would be considered effective if it is used by larvae and adults for shelter or if it can be used for laying eggs.

## 2. Materials and Methods

### 2.1. Study Area

The Ascotán salt flat is located in the highlands of the Antofagasta Region, Chile, at 3720 m a.s.l., on the border with Bolivia. It encompasses 243 km^2^, of which 18 km^2^ are covered by lagoons. Only 0.035 km^2^ correspond to small lagoons associated with eleven springs, which contain waters of relatively lower salinity and support floodplains along the lagoon outflows [18].

The habitat enhancement program was implemented in spring 11 (V11), located at the southeastern end of the Ascotán salt flat, and has an approximate area of 5.1 ha. From a habitat and physicochemical perspective, V11 has a similar substrate, depth, aquatic vegetation cover, pH, and turbidity to two other natural springs (V6 and V7) that host *Telmatobius*. However, it recorded higher temperatures and lower levels of total dissolved solids and conductivity, differences which may result from the artificial recharge (Table S4 in Supplementary Materials).

To assess the effectiveness of the habitat enhancement, nine monitoring campaigns were conducted between 2022 and 2024: M1, August 2022 (winter); M2, September 2022 (winter); M3, October 2022 (spring); M4, March 2023 (summer); M5, June 2023 (fall); M6, August 2023 (winter); M7, November 2023 (spring); M8, March 2024 (summer); and M9, June 2024 (fall).

### 2.2. Implementation of Refuges

**Figure 1:**
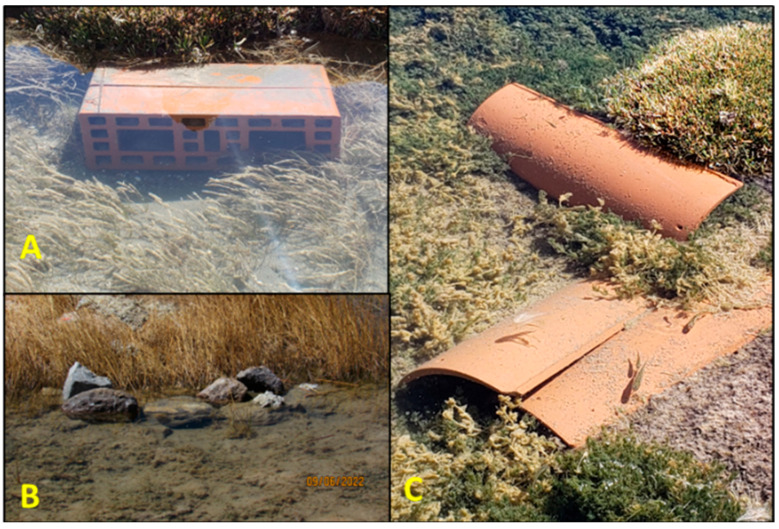
Types of artificial refuges implemented in Spring 11. (**A**) Bricks, (**B**) rock piles, and (**C**) clay tiles.

In June and July 2022, various types of artificial refuges were installed in the V11 spring. The refuges’ locations were selected to ensure an even distribution throughout the spring, in areas where aquatic vegetation occurs (Figure 1). Refuges included bricks with hollows 29 cm long, 14 cm wide, and 7 cm high (*n* = 36 individual bricks); clay tiles 39 cm long, 20 cm wide, and 17 cm high (*n* = 99; arranged in groups of three); and rock piles created from rocks present on site (*n* = 4). Altogether, the refuges were distributed across an area of 1087 m^2^.

### 2.3. Refuge Monitoring

For logistical and safety reasons, monitoring was conducted between 9:00 AM and 4:00 PM. During each two-day monitoring session, three researchers carefully checked the refuges to record the presence of *Telmatobius* larvae, juveniles, and adults. Any individuals present were temporarily captured and placed in a 27.68 × 27.94 cm plastic Ziploc^®^ bag filled with water from the capture site. We identified recaptured individuals by detecting the presence of a passive integrated transponder (PIT tag). Individuals without a PIT tag, with a snout–vent length ≥ 45 mm, were subcutaneously implanted with one (8 × 1.4 mm, 134.2 kHz; Biomark Inc., Boise, ID, USA). Biometric measurements were obtained from all captured individuals, which were returned to the capture site within approximately 5 min of capture. Special care was taken to ensure the animals remained in the refuge after release. The percentage of the refuge occupancy was calculated by dividing the number of animals registered in the refuges by the total number of refuges, multiplied by 100.

### 2.4. Demographic Analysis

Only adults were considered in the population model. The population size in adults was estimated based on capture and recapture data using the CAPTURE module available in R [32]. The selection of the best model was based on the Akaike information criterion (AIC). Only a numerical record was kept for the larvae without any marking, and their age structure was assessed according to Gosner’s developmental stages [33]. For this, the monitoring records were grouped into prometamorphic (G1; stages 23 to 26), premetamorphic (G2; 27 to 41), and metamorphic climax (G3; 42 to 46) [34,35]. Generalized linear models (GLMs) with Binomial distribution and logit link function were used to test the relationships between the proportions of the different larval classes, with years and their seasons [36]. We used a stepwise backward selection approach and applied Akaike’s information criterion to select the best model [37], using the R package version 4.5 [38].

### 2.5. Biometric Measurements

Sex determination based on secondary sexual characteristics was feasible exclusively in adults, characterized by nuptial patches on the toes and chest of males, rounded body shape in gravid females (length of the abdomen relative to body size and legs), and nasal morphology (females exhibit a rounded nose, whereas males possess an acuminate nose). In adults and subadults, body length (snout–vent length in millimeters; SVL) and mass (grams) were recorded, and a body condition index (BCI) was determined using the residuals of the linear regression of the logarithm of mass and logarithm of the SVL [39]. The non-parametric Kruskal–Wallis test (H) was used for comparisons between sexes and seasons, as the data failed to satisfy the normality assumption of the Shapiro–Wilk test. Significant differences (*p* < 0.05) among means were evaluated with Dunnet’s a posteriori test performed in Infostat [40]. Larval length (total length in millimeters) and mass (grams) were recorded for the different groups (G1, G2, and G3) according to Gosner (1960) [33].

All individuals were handled considering a biosecurity protocol for emerging diseases [41].

## 3. Results

### 3.1. Refuge Occupancy

Individuals were only recorded in the brick refuges and rock piles at the beginning of the study; after the third survey, the holes in the bricks became clogged with sediment, and the rock piles were only occasionally occupied. Therefore, we only analyzed data obtained from the tile refuges.

**Figure 2:**
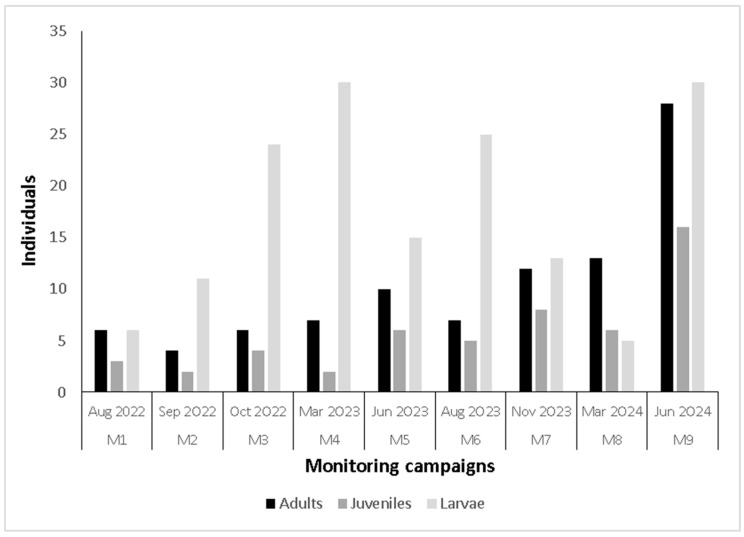
Abundance of *Telmatobius philippii* life stages (adults, juveniles, and larvae) captured in artificial tile refuges during nine monitoring campaigns.

The numbers of larvae, juveniles, and adults captured in the tile refuges are shown in Figure 2. *Telmatobius* were documented utilizing the refuges in all monitoring sessions and increased over time through the end of the study. The number of adults captured in the tile refuges ranged from four individuals during the M2 monitoring session to 28 during the M9 session. Juveniles showed a similar pattern increasing from two individuals in M2 to sixteen in M9. The number of larvae ranged from 5 (M8) to 30 individuals (M9). The peak larvae abundance was documented during four monitoring campaigns with 24 larvae recorded during M3, 30 during M4, 25 during M6, and 30 during M9. The fewest number of larvae were recorded during M1 (n = 6) and M8 (n = 5). Additionally, egg clutches and early-stage larvae (G1) were observed in one of the refuges during the M6 (winter) and M7 (spring) campaigns.

The occupancy rates of individual refuge sites varied as follows: for adults, from 4% (M2) to 28.3% (M9); for juveniles, from 2% (M2) to 16% (M9); and for larvae, from 5% (M1) to 30% (M9). The average occupancy rate across all 33 tile refuge sites and monitoring campaigns ranged from 15% (M1) to 75% (M9) (Figure S1 in Supplementary).

### 3.2. Population Aspects of Larvae

Across all monitoring campaigns, a total of 165 larvae were captured, including individuals from all three age classes (G1, G2, and G3) (Table S1 in Supplementary). G2 larvae were proportionally the most abundant in every survey. According to the GLM, there were significant differences in the proportions among age groups (*p* < 0.05), favoring G2, but no differences were detected between years or seasons (Tables S2 and S3 in Supplementary Materials).

### 3.3. Population Aspects of Adults and Juveniles

A total of 65 adults and 27 juveniles were captured during this study. Considering only adults, there were 34 recapture events, involving 21 individuals caught only once, 5 on two occasions, and 1 on three occasions. Two population models were determined to have the best fit: M(t), with temporal heterogeneity in the likelihood of recaptures, and Mth Chao (LB), with temporal and individual heterogeneity. Both models resulted in an estimated population size of 97 ± 11 individuals (Table 1).

### 3.4. Biometric Aspects

**Figure 3:**
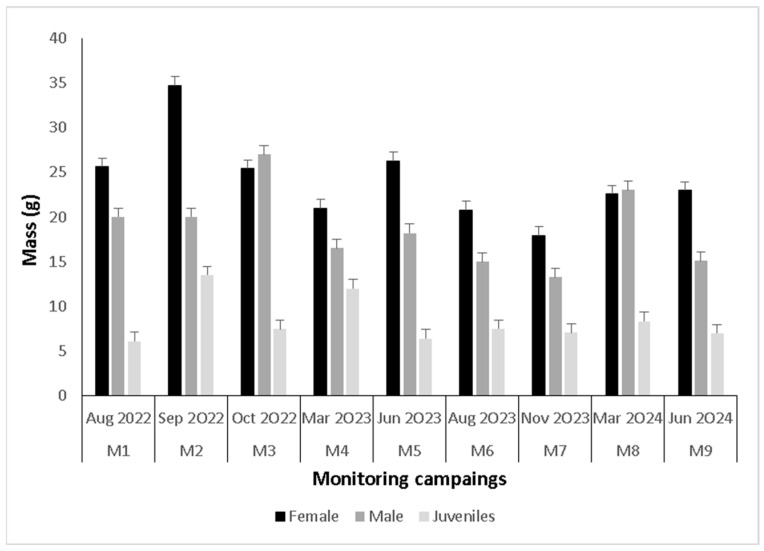
Body mass in grams for adults (females and males) and juveniles *Telmatobius philippii* by monitoring campaign.

**Figure 4:**
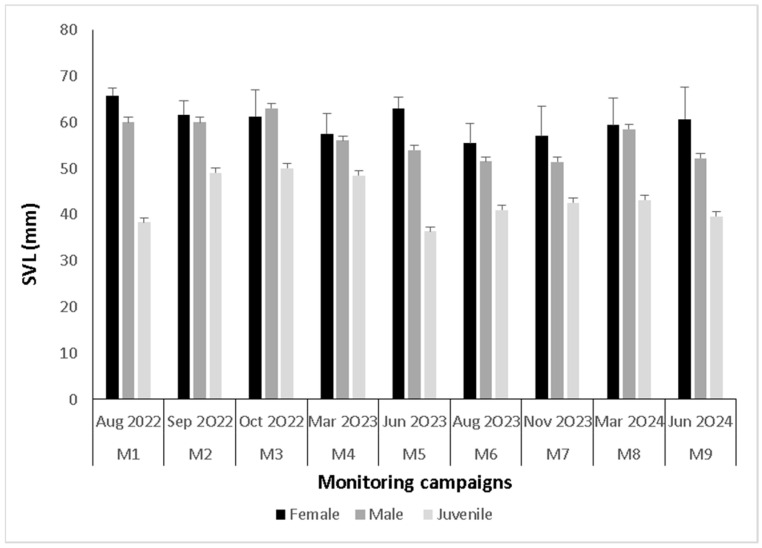
Body length (SVL) in millimeters for adults (females and males) and juveniles *Telmatobius philippii* by monitoring campaign.

For adults (including recaptures), females (23.75 ± 7.5 g, n = 51) were heavier than males (16.11 ± 5.5 g, n = 39) (H = 28.1, *p* < 0.01), and there were no differences between seasons for females (H = 12, *p* = 0.15) or males (H = 14.7, *p* = 0.07) (Figure 3). Females (60.3 ± 5.7 mm, n = 51) were longer than males (53.3 ± 5.1 mm, n = 39) (H = 26.3, *p* < 0.01). Regarding seasons, differences were observed only in females (H = 16.6, *p* = 0.03), where in M6 (winter) they were smaller than those in M1 (winter) (Figure 4). For subadults, no differences were recorded between seasons for either body mass (7.5 ± 3.7, n = 50. H = 7.96, *p* = 0.4) or length (41.7 ± 7.5, n = 50, H = 10.58, *p* = 0.22).

**Figure 5:**
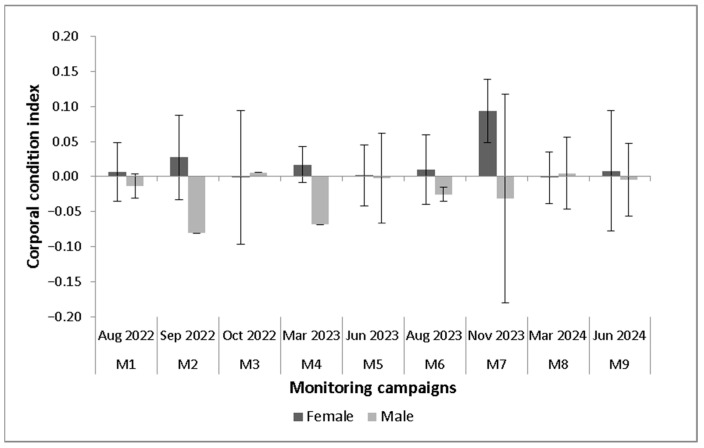
Variation in *Telmatobius philippii* body condition index (adults) between sexes and by monitoring campaign.

Regarding body condition indices (Figure 5), there were no differences between sexes (H = 0.13, *p* = 0.9) or between seasons for females (0.11 ± 0.02, H = 14, *p* = 0.08) and males (0.11 ± 0.02, H = 3.41, *p* = 0.9). However, it was generally observed that females presented a higher body condition value than males in monitoring campaign M1 through M7. In M2, there was a peak in the value of females attributed to the presence of a single large gravid female, and in M8 and M9, there was an increase in body mass for both sexes. For females, ICC values were low (less than zero) in M1, M3, and M7; for males, values were low throughout most of the study (M1, M2, M4, M5, M6, and M7).

In larvae there were significant differences in body mass across age classes (G1 3.65 ± 0.7, n = 26; G2 9.04 ± 0.3, n = 120; and G3 6.7 ± 0.9, n = 13), with G2 exhibiting the highest values (H = 38.9, *p* < 0.01). Seasonal differences were only significant for the G2 age class (H = 28.4, *p* < 0.01), with lower values during M8 (summer) and M9 (autumn) compared to M2 (winter) and M7 (spring) (Figure S2 in Supplementary Materials). There were differences in body length among age classes (H = 52.7, *p* < 0.01), with G2 being larger, and there were no differences between G1 and G3 (G1 56.4 ± 2.7, n = 26; G2 81.7 ± 1.25, n = 120; and G3 50.2 ± 3.8, n = 13). There were seasonal differences in the G2 age class (H = 38.1, *p* < 0.01), where individuals captured during the M3, M4, M8, and M9 monitoring sessions were smaller than those captured during the M2, M6, and M7 sessions and in the G3 age class (H = 9.6, *p* = 0.04) where M6 individuals were smaller than in M4 and M5 (Figure S3 in Supplementary).

## 4. Discussion

Artificial refuges can provide safe spaces for animals to breed, hibernate, or take refuge [42]. In fish, artificial structures have promoted recruitment [43,44,45], provided protection from predators [46,47,48], and provided shade [49,50]. Studies on the use of artificial refuges by amphibians are scarce [42], with limited evidence of efficacy [51], and have primarily focused on arboreal and terrestrial species [42]. For *Telmatobius*, the use of artificial refuges has been investigated during ex situ breeding programs [52,53,54,55,56].

In our study, clay tiles were the only type of artificial refuge that remained effective throughout the monitoring campaign. One factor reducing the effectiveness of brick refuges utilized in ex situ breeding programs has been obstruction by sediments [56], similar to our observations during this study, which we attribute to sluggish water flow and floodplain sedimentation in V11. The clay tiles were colonized by *Telmatobius* from the first monitoring session, and the occupancy rate increased progressively over time, reaching 75% by the end of the study. The refuges were used by all age classes, including larvae, which indicated successful reproductive activity in the spring.

Lobos et al. (2018) only recorded an average of 3.7 larvae in a 5 m^2^ sampling area within V11 and attributed the low values to previous hydrological and physical disturbance of the V11 lagoon and floodplain [26]. Low larval densities were also documented during the FNDR study project (2021–2022), which documented an average of 0.75 larvae in the same sampling area [31]. Larval densities in the tile refuges installed for this study ranged from 5 to 30 larvae, which, in conjunction with the observation of eggs and prometamorphic larvae, demonstrates the effectiveness of these artificial refuges in increasing this population parameter. The occupancy value of the refuges could have been even higher, but we noted that the refuges located upstream of the gauging station had a lower occupancy. The gauging station has created a narrowing of the lagoon, creating a channel that serves as a physical barrier to the upstream passage of amphibians. However, one specimen captured and tagged downstream from the gauging station moved upstream approximately 120 m and was subsequently recaptured above the station later. Although this individual could have traveled upstream through the water, there are photographs of the Ascotán community depicting frogs moving by land, a phenomenon not previously documented for any Chilean *Telmatobius* species [26,57,58].

The biometric data recorded in this study are consistent with the information known for this species [26], with sexual dimorphism favoring females (weights and sizes) and larvae that likely require more than one year to develop fully. Population models estimated the adult *Telmatobius* population within the habitat enhancement area, which constitutes a significant portion of the optimal habitat for amphibians in V11 due to the water depth (15 to 35 cm), at 97 ± 11 individuals. This represents a significant increase from the population estimate of 18 adults by Lobos et al. (2018) [26], although their study area was slightly smaller than the current study. Among the recaptures, an individual previously marked in the FNDR Project (November 2021) was caught 31 months later in M9. In addition, there was a record of a gravid female during M2 (53 gr) who was recaptured in M3 when she had already expelled her eggs (38 gr).

However, the population estimate derived from the current study (97 ± 11 individuals) is still relatively small and remains vulnerable to stochastic natural events and anthropogenic threats. Additionally, while there was an increase in the number of larvae (reproduction) documented in V11 during the study and BCI values (a proxy for individual health) were generally relatively stable, BCI values for male *Telmatobius* were negative in five of the nine monitoring sessions and in three sessions for females, irrespective of the season. The larval development was highly heterogeneous. The masses and sizes of the G2 group were greater than their counterparts, reflecting the long larval development times previously reported for high Andean anurans [59].

Even though our study had certain methodological limitations, such as the lack of experimental replicates (the lagoon is small, with an effective habitat for *Telmatobius* of 0.26 ha), the results are encouraging for *Telmatobius* conservation in areas impacted by human activities. Globally, amphibians are under significant threat from a variety of factors, including disease, climate change, and human-caused habitat loss [1,2,11], which pose major challenges for local conservation efforts. In this sense, field experiences such as this study are fundamental for strengthening future conservation programs.

## 5. Future Directions

Moving forward, the habitat enhancement program requires long-term commitment. Essential future efforts include managing aquatic vegetation to control eutrophication; maintaining artificial refuges (during this study, 20% of the tiles had to be replaced annually due to climatic factors and human intervention); and ensuring strict biosecurity protocols for all personnel to prevent the introduction of pathogens, as none have been detected to date.

Furthermore, it is also essential to maintain the monitoring program for groundwater levels—particularly once water extraction ceased, which is permitted until 2029—to reassess this population at least annually and compare it with natural populations present in other springs, to promote the protection of natural springs that still harbor this species, and to strengthen environmental education programs with the local communities.

## 6. Conclusions

The implementation of artificial refuges, particularly clay tiles, proved to be an effective conservation measure for *Telmatobius philippii* in the Ascotán salt flat. The progressive increase in refuge occupancy, reaching 75% by the end of the study, along with the recording of egg clutches and early-stage larvae, confirms that the availability of refuges was a limiting factor that was successfully mitigated through this intervention.

Despite the observed success, the population of *T. philippii* in spring 11 remains small (approximately 97 adults) and vulnerable to natural and anthropogenic threats. Long-term efforts are recommended, including refuge maintenance, aquatic vegetation management, continuous monitoring, and environmental education, to consolidate the recovery of this population in the face of increasing anthropogenic pressure.

## Figures and Tables

**Table 1:** Results of the capture and recapture models. (Abundance = estimated population abundance of *Telmatobius philippii* in Spring 11, d.f. = degrees of freedom, AIC = Akaike’s information criterion value, and * = best fit of the model).

Models	Abundance	Standard Error	Deviance	d.f.	AIC
M0	100.1	11.6	111.0	509	186.9
Mt *	96.5	10.8	75.8	501	167.7
Mh Chao LB	100.1	11.6	111.0	509	186.9
Mh Darroch	90.5	18.1	110.7	508	188.6
Mh Gamma 3.5	87.2	21.1	110.7	508	188.6
Mth Chao LB *	96.5	10.8	75.8	501	167.7
Mth Darroch	90	17.7	75.7	500	169.5
Mth Gamma 3.5	87.6	21.4	75.7	500	169.5

## Data Availability

*Telmatobius philippii* are listed as Endangered in Chile; therefore, to prevent unnecessary harm, the exact geographic coordinates of individuals cannot be publicly shared. However, reasonable requests for research data can be made to the corresponding author for consideration.

## Supplementary Materials

The following supporting information can be downloaded at https://www.mdpi.com/article/10.3390/ani15213156/s1: Table S1: Number of larvae recorded in refuges, by season, and Gosner age groups; Table S2: Comparison of generalized linear models for the evaluation of the determining factors between the proportions of the different larval classes (G1, G2, and G3), with years and seasons. The model with the lowest Akaike information criterion (AIC) was chosen as the best and is shown in bold. R^2^ coefficient of determination; Table S3: The best model determining relationships between the proportions of the different larval classes with years and seasons. B is the coefficient of multiple regression, Z is the Z-test value, and *p* is the probability under the null hypothesis. Values in bold indicate significance; Table S4: Microhabitat variables recorded for *Telmatobius philippii* in three springs at Ascotan in northern Chile; Figure S1: Percentage of refuge sites occupied by adults, juveniles, and larvae across all monitoring campaigns; Figure S2: Mass in grams for larval age classes by monitoring campaign; and Figure S3: Length in millimeters for larval age classes by monitoring campaign.
