# Supplementary material for: Conservation in the Andean Highlands of South America: A Habitat Enhancement Plan for Tematobius philippii, a Critically Endangered Species in the Ascotán Salt Flat in Chile
Source: Animals (Basel). 2025 Oct 30;15(21):3156. doi: 10.3390/ani15213156 (PMC12607360; doi:10.3390/ani15213156)
Supplement: Supplementary file 1 [file animals-15-03156-s001.zip › animals-3937900-supplementary.pdf]

Supplementary Materials

Table S1. Number of larvae recorded in refuges, by season and Gosner age groups.

Campaigns	Date	G1	G2	G3	Total
M1	Aug 2022	5	1	0	6
M2	Sep 2022	1	9	0	10
M3	Oct 2022	9	30	0	39
M4	Mar 2023	5	22	3	30
M5	Jun 2023	2	12	1	15
M6	Aug 2023	3	22	1	26
M7	Nov 2023	0	9	1	10
M8	Mar 2024	1	4	0	5
M9	Jun 2024	0	26	4	30
Total		21	134	10	165

Table S2. Comparison of generalized linear models for the evaluation of the determining factors between the proportions of the different larval classes (G1, G2, and G3), with years and seasons. The model with the lowest Akaike information criterion (AIC) was chosen as the best and is shown in bold. R^2 coefficient of determination.

Models	AIC	R^2
Variables		
Gosner G2+ Gosner G3+ Years + Autumn + Spring + Summer	23.9	0.7
Gosner G2 + Gosner G3 + Years	17.9	0.7
Gosner G2+ Gosner G3	**15.9**	0.7

Table S3. The best model determining relationships between the proportions of the different larval classes with years and seasons. B is the coefficient of multiple regression, Z is the Z-test value, and p is the probability under the null hypothesis. Values in bold indicate significance.

	Model		
Variables	B	Z	p
Intercept	-1.84	-0.03	0.97
Gosner G2	2.09	2.05	**0.04**
Gosner G3	-0.13	-0.09	0.92

Table S4. Microhabitat variables recorded for *Telmatobius philippii* in three springs at Ascotan in northern Chile.

Parameters	V06	V07	V11
Temperature (Cº)	14,5 ± 0,72	17 ± 0,64	22 ± 4,2
EC (mS cm^{-1})	4,4 ± 0,07	4,6 ± 0,1	2,9 ± 0,18
OD (mg L^{-1})	8,6 ± 2,01	6,4 ± 1,37	8,4 ± 2,37
pH	8,2 ± 0,29	7,8 ± 0,26	7,9 ± 0,43
Dissolved solids (ppm)	2,2 ± 0,05	2,3 ± 0,05	1,5 ± 0,1
Turbidity (NTU)	0 ± 0	0 ± 0	0,91 ± 1,07
Aquatic vegetation cover (%)	87,5 ± 4,33	75 ± 11,18	82,5 ± 14,79
Substrate	Mud/gravel	Mud/gravel	Mud/gravel
Depth (cm)	30	22	18

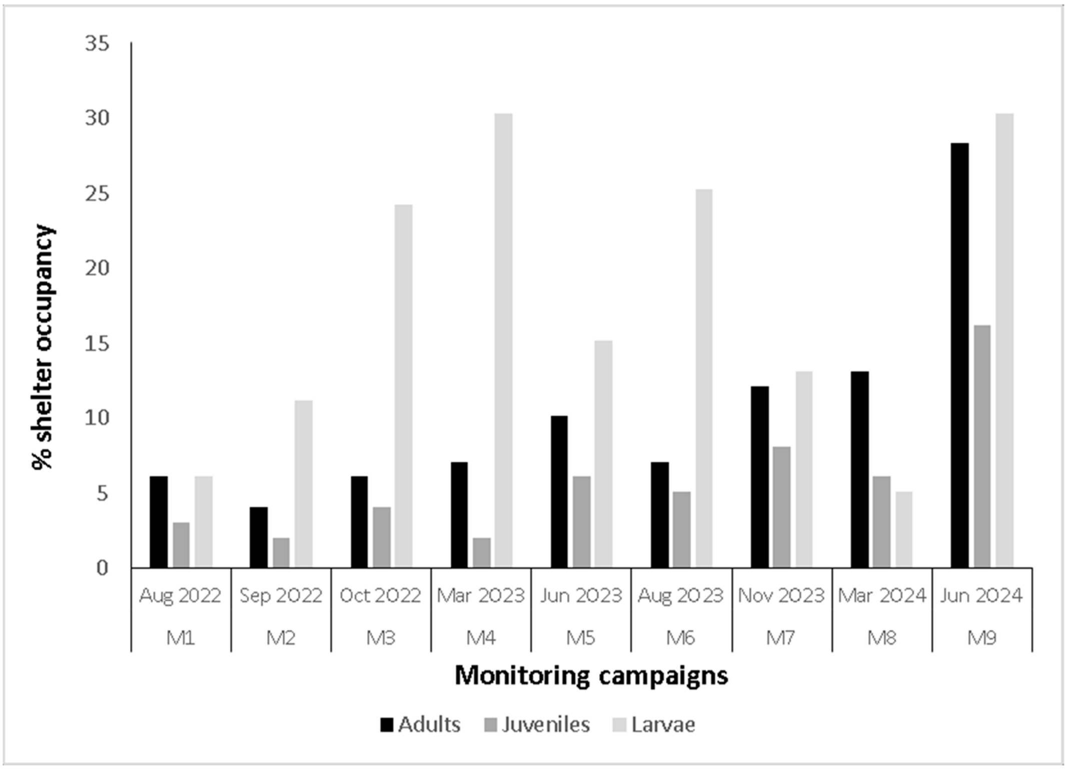

Figure S1. Percentage of refuge sites occupied by adults, juveniles, and larvae across all monitoring campaigns.

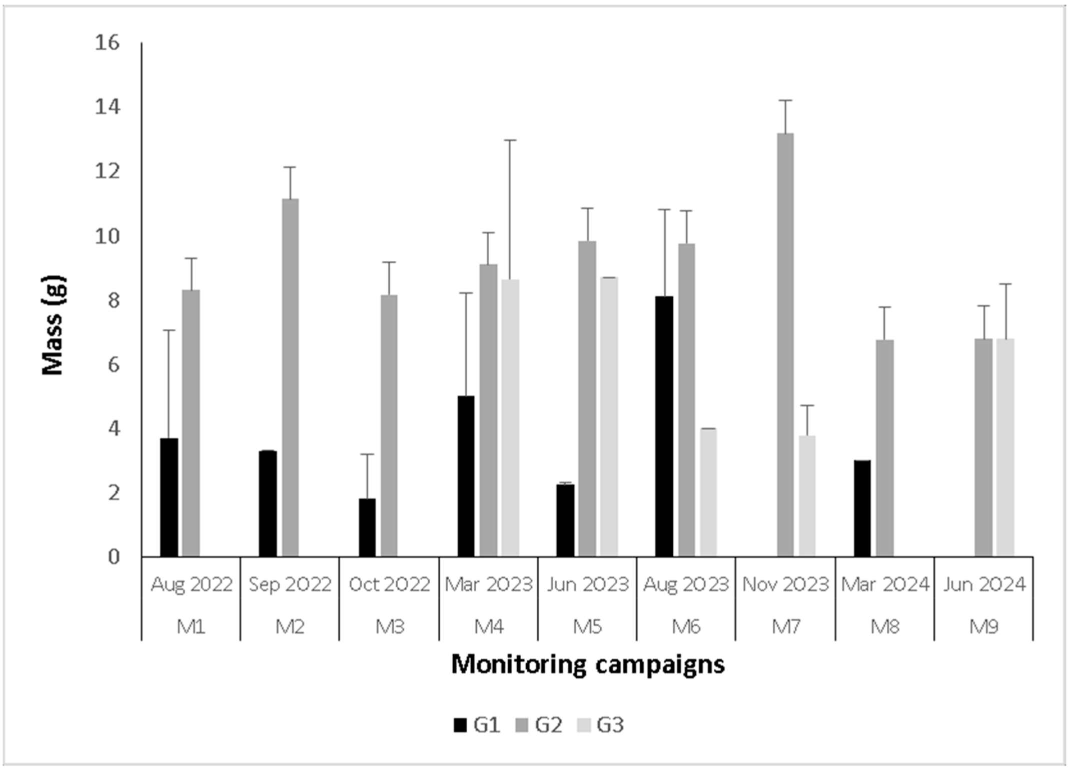

Figure S2. Mass in grams for larval age classes by monitoring campaign.

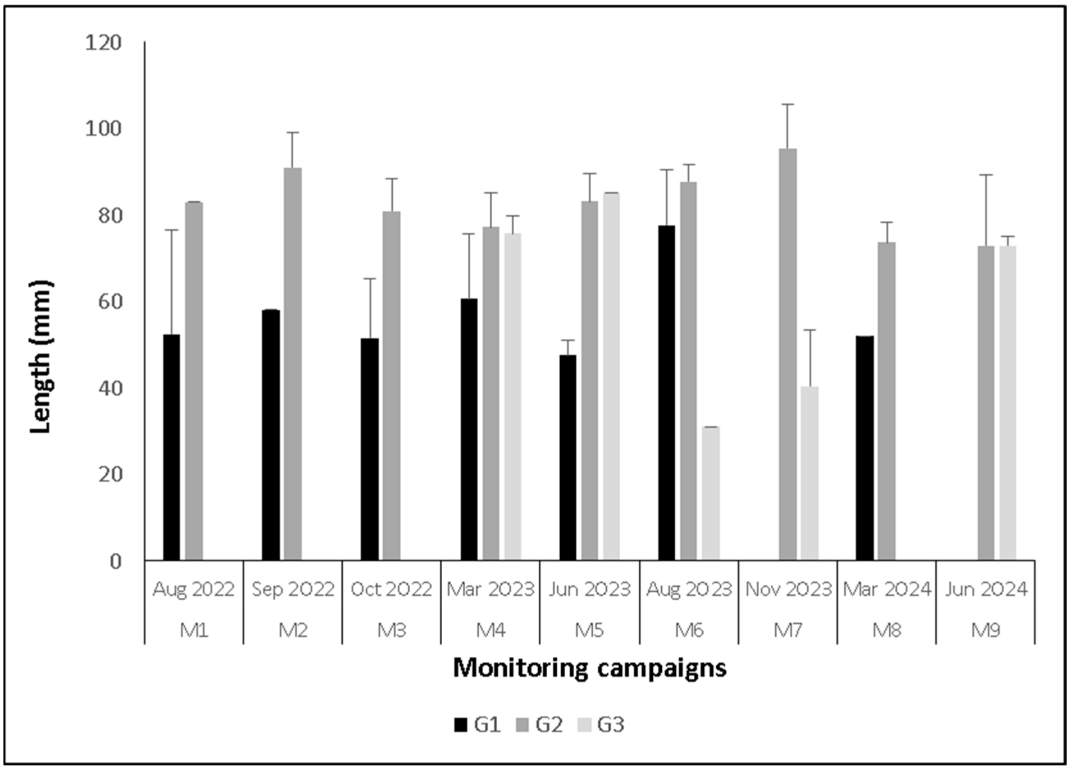

Figure S3. Length in millimeters for larval age classes by monitoring campaign.
